# Nutrient cycling potential within microbial communities on culturally important stoneworks

**DOI:** 10.1111/1758-2229.12707

**Published:** 2018-12-25

**Authors:** Elisabetta Zanardini, Eric May, Kevin J. Purdy, J. Colin Murrell

**Affiliations:** ^1^ School of Life Sciences University of Warwick Coventry UK; ^2^ Department of Science and High Technology University of Insubria Como Italy; ^3^ School of Biological Sciences University of Portsmouth Portsmouth UK; ^4^ School of Environmental Sciences University of East Anglia Norwich UK

## Abstract

Previous studies on microbes associated with deterioration of cultural heritage (CH) stoneworks have revealed a diverse microbiota adapted to stresses such as low nutrients, aridity and high salinity, temperatures and radiation. However, the function of these pioneer microbial communities is still unclear. This study examines bacterial and archaeal diversity in exfoliated and dark encrustation sandstone from Portchester Castle (UK) by 16S rRNA and functional gene analyses. Bacterial and archaeal communities from the exfoliated sites were distinctly different from the dark encrustation. Detected genera were linked to extreme environmental conditions, various potential functional roles and degradation abilities. From these data it was possible to reconstruct almost complete nitrogen and sulfur cycles, as well as autotrophic carbon fixation and mineral transformation processes. Analysis of RNA showed that many of the detected genera in these nutrient cycles were probably active *in situ*. Thus, CH stonework microbial communities are highly diverse and potentially self‐sustaining ecosystems capable of cycling carbon, nitrogen and sulfur as well as the stone biodeterioration processes that lead to alterations such as exfoliation and corrosion. These results highlight the importance of diversity and internal recycling capacity in the development of microbial communities in harsh and low energy systems.

## Introduction

Cultural heritage (CH) objects, including stoneworks, are affected by chemical, physical and biological processes which can modify their structure and composition; bacteria, archaea and fungi cause chemical, mechanical and aesthetic damage to CH stone materials (Sorlini *et al*., 1987; Zanardini *et al*., 2011; Warscheid and Braams, 2000; Ranalli *et al*., 2009; May, 2010). This damage can be caused by low biomass biofilms forming on the stone, including growth of filamentous organisms such as actinomytes and fungi, which can physically penetrate into the stone (Suihko *et al*., 2007; Scheerer *et al*., 2009; Sterflinger, 2010), by acidifying bacteria such as sulfur oxidizers and nitrifiers, which can cause corrosion (Mansch and Bock, 1998; Abeliovich, 2006), and phototrophic or pigmented microorganisms, which discolour and stain the stone (Crispim and Gaylarde, 2005; Cappitelli *et al*., 2009).

The microbial communities involved in the biodeterioration of CH stoneworks have been investigated using microscopic, culture‐dependent and ‐independent methods both in indoor and outdoor environments. Investigations of the prokaryotic and eukaryotic community structure based on small subunit rRNA genes showed in some cases a high biodiversity of the these communities, with members of phyla from Actinobacteria, Proteobacteria, Chloroflexi and from the Archaea linked with alterations such as discolorations, patinas and crusts (McNamara and Mitchell, 2005; Ranalli *et al*., 2009; Scheerer *et al*., 2009; May, 2010; Ettenauer *et al*., 2014; Zanardini *et al*., 2016). High‐throughput sequence analyses of microbial communities on rocks and stoneworks have shown that these communities are more complex than previously thought and have highlighted the difficulties in analyzing such samples effectively (Gutarowska *et al*., 2015; Chimienti *et al*., 2016; Adamiak *et al*., 2017). Wu *et al*. (2015) detected greater than 600 bacterial species‐level operational taxonomic units (OTUs) from samples from a limestone cave in China. A number of OTUs related to putative cave‐lineages from the gamma‐proteobacteria and actinobacterial phyla were detected along with OTUs related to bacterial clades capable of autotrophic carbon fixation (e.g., *Thioprofundum*) and ammonia oxidation (e.g., *Nitrospira*). Analysis of CH samples exposed to the weather also detected complex communities of phototrophs such as algae and cyanobacteria and heterotrophs from Proteobacteria and Actinobacteria (Gutarowska *et al*., 2015).

Studies on the function and activity of communities involved in the biodeterioration processes of rocks and stoneworks are rare and there is a need to understand potential and active functions to determine possible deterioration processes within these CH communities and are relevant to other extreme and low energy systems. Distinct differences between the bacterial communities in natural caves with prehistoric paintings in Spain have been detected using DNA and RNA‐based molecular fingerprinting and cloning approaches (Portillo *et al*., 2009).

Microbial communities cycle nutrients such as carbon, nitrogen and sulfur (Falkowski *et al*., 2008) which are particularly important when establishing a viable community on a harsh environment like stone where nutrients from other biological sources are likely to be sparse. Thus, it is reasonable to suppose that to establish a sustainable community on stone, colonizing bacteria need to cycle carbon, nitrogen and sulfur effectively to establish a persistent community. Analysis of functional genes associated with sulfur cycling (*soxB*, *dsrA* and *apsA*) have been detected on limestone tombstones indicating the potential for a complete sulfur cycle at that site (Villa *et al*., 2015).

In our previous work (Zanardini *et al*., 2016) we investigated bacterial and archaeal community structure and diversity in exfoliated sandstone at Portchester Castle (UK) using 16S rRNA gene profiling by DGGE and direct isolation of bacteria. These studies suggested that many of the bacteria and archaea that have colonized the Castle are closely related to strains adapted to ‘extreme’ environments, especially high salinity (e.g., *Halococcus*) and UV radiation (*Truepera radiovictrix*), low water availability (*Pontibacter akensuensis*) and high temperatures (*Microbacterium*), but the study provided only a limited molecular analysis of the microbial communities. Following on from this previous study in which we showed colonization of decayed stone from Portchester Castle here we have used DNA‐ and RNA‐targeted high throughput sequencing along with targeted functional gene analyses to determine the diversity, potential activity and nutrient cycling functionality of microbial communities on these culturally important stoneworks.

## Materials and methods

### *Portchester Castle and altered sandstone sampling*

Portchester Castle is a medieval castle built on a former Roman fort at Portchester, Hampshire, UK. Information on site, meteorological conditions, green sandstone composition and characteristics are reported in our previous study (Zanardini *et al*., 2016). In June 2009 samples of green sandstone were taken from two distinct regions on a window in Richard II's banqueting room within the keep of the castle (Supporting Information Fig. S1A). Sites 1 and 2, respectively, to the lower right and left of the window, both showed extensive exfoliation. Site 3, to higher left of the window, was different as it showed a dark encrustation (Supporting Information Fig. S1B). Three replicate samples were aseptically taken by scraping the altered stone material using a sterile scalpel from a surface area of 10 cm^2^ of each site. The samples were kept at 4°C during transportation to the University of Warwick (UK). English Heritage authorized the sampling regime at Portchester Castle.

### *DNA and RNA extraction from altered stone samples*

DNA for PCR of functional genes was extracted from 0.5 g crushed stone samples using the FastDNA® SPIN Kit for soil (MP Biomedicals, USA) as reported previously (Zanardini *et al*., 2016). In additon, in order to analyze samples for expression as well as presence of 16S rRNA and functional genes, DNA and RNA were extracted from the same 0.5 g crushed stone samples using the hydroxyapatite spin column method (Purdy, 2005).

### *PCR and RT‐PCR amplification*

Extracted RNA was DNAse treated using RQ1 RNase‐Free DNase (Promega, UK) to remove any trace DNA prior to reverse transcription. First‐Strand cDNA synthesis was performed using Invitrogen SuperScript III Reverse Transcriptase and suitable controls as per manufacturer's instructions (Thermo Fisher, UK). PCR was used to amplify genes from DNA and cDNA from the three sites targeting the genes shown in Table 1. PCR primers used are presented in Supporting Information Table S1.

**Table 1:** Genes analyzed by PCR and RT‐PCR from all three Portchester Castle samples.

	Pyrosequenced samples							Cloned samples		
	16S rRNA				*amoA*				*cbbL*	
Site	Bacteria		Archaea		Bacteria		Archaea	*nirK*	Green	Red
	DNA	RNA	DNA	RNA	DNA	RNA^a^	DNA	DNA	DNA	DNA
1	**Y**	**Y**	**Y**	**Y**	**Y**	**Y**	**Y**	–	–	–
2	**Y**	**Y**	**Y**	**Y**	**Y**	–	**Y**	**Y**	**Y**	**Y**
3	**Y**	–	**Y**	–	–	–	–	–	–	–

a Bacterial RT‐PCR product also cloned and sequenced; − indicates samples not analyzed.

### *Pyrosequencing methods and analysis*

Pyrosequencing was performed at Research and Testing Laboratory (Lubbock, TX, USA) using tagged amplicon methods as described previously (Dowd *et al*., 2008) modified for titanium chemistry (Roche, Branford, CT, USA). Briefly, 20 cycles of PCR were utilized (94°C for 30 s, 50°C for 30 s and 72°C for 40 s) with a final extension at 72°C for 10 min to incorporate the linkers and tags. A 200 flow Titanium sequencing run was performed according to Roche protocols with amplicon signal processing. Following sequencing and image processing, the sequences were binned into individual multi‐fasta files using custom software and used for data analysis. 16S rRNA sequences were clustered at 97% and *amoA* sequences were clustered at 90% after quality control and chimera checking (Dowd *et al*., 2008). OTUs were identified using BLASTn (Altschul *et al*., 1990) against a custom database derived from the RDP‐II database (Maidak *et al*., 2001) and GenBank.

### *PCR product cloning, sequencing and phylogenetic analysis of functional genes*

PCR and RT‐PCR products from bacterial and archaeal *amoA*, *nirK*, green‐like and red‐like RuBisCO genes from site 2 were cloned into pGEM Vector (pGEM®‐T Easy Vector Systems, Promega, UK) as per manufacturer's instructions. For each sample and target‐gene at least 20 clones were randomly selected and analyzed by RFLP (Britschgi and Fallon, 1994) using *Bsu*RI (bacterial *AmoA* gene), *Rsa*I (archaeal *AmoA* gene), *Mbo*I (*nirK* gene) and *Hpa*II (RuBisCO). Representative clones from each distinct RFLP group were sequenced using M13 vector primers at the Molecular Biology Services, University of Warwick.

### *Phylogenetic analyses of cloned functional genes*

Cloned sequences were initially analyzed using BLAST (Altschul *et al*., 1990). Sequences identified as similar to the target gene were aligned with related sequences from reference strains and environmental clones using ClustalX (Version 1.8) (Thompson *et al*., 1997). Neighbour‐Joining phylogenetic trees were generated using MEGA4 (Tamura *et al*., 2007).

### *Sequence accession numbers*

All sequences have been submitted to GenBank. Accession numbers; Bacterial *amoA* gene MG928324–MG928330; Archaea *amoA* gene MG928331–MG928347; *nirK* gene MG928348–MG928357; green Rubisco gene MG928358–MG928367 and Red Rubisco gene MG928368–MG928384. Pyrosequence datasets have been submitted to the Short Read Archive under accession numbers SAMN09622501–SAMN09622510.

## Results and discussion

**Figure 1:**
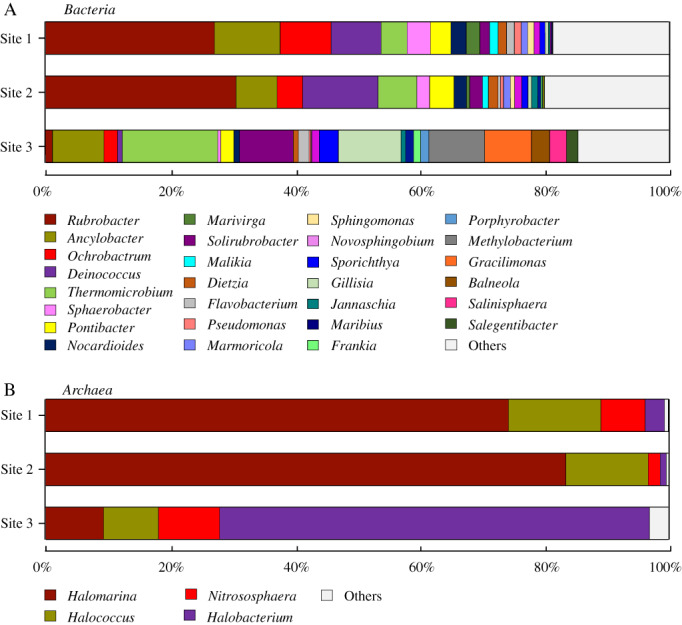
Genus‐level identification of (A) bacterial and (B) archaeal communities from altered stoneworks, Portchester Castle.

High throughput sequencing analysis of 16S rRNA gene fragments amplified from DNA extracted from three samples of altered stone from Portchester Castle showed highly diverse bacterial communities, with between 108 and 180 bacterial genera detected in the three samples analyzed (Fig. 1A; Supporting Information Table S2). Archaeal diversity was far more limited with between 4 and 7 genera detected in each sample and these were dominated by members of the halophilic *Halobacteriales* (Fig. 1B; Supporting Information Table S3). Many of the detected bacterial and archaeal genera can be linked to the ability to tolerate extreme environments, as well as the capacity to perform critical steps in the cycling of carbon, nitrogen and sulfur (see Supporting Information Tables S2 and S3). The prevalence of *Actinobacteria, Alphaproteobacteria, Bacteroidetes, Deinococcus‐Thermus* and *Chloroflexi* phyla and halophilic *Archaea* confirms data presented previously from DGGE and isolate analyses from the samples used in this study (Zanardini *et al*., 2016) and from previous studies on rock and stone habitats which also highlighted a number of these phyla (Sorlini *et al*., 1987; Zanardini *et al*., 2011; Ettenauer *et al*., 2014; Marnocha and Dixon, 2014; Gutarowska *et al*., 2015; Wu *et al*., 2015).

The sites showed distinct community differences (Fig. 1), with the exfoliating sites 1 and 2 very similar to each other while the dark encrustation at site 3 had distinctly different bacterial and archaeal communities (Supporting Information Tables S2 and S3). Overall, the 16S rRNA gene analysis revealed sequences from the same genera of all but one of the previously reported isolates (an *Enterococcus*; Zanardini *et al*., 2016) as well as many bacterial genera that were not detected in the previous study (Supporting Information Table S2). Genera associated with survival under extreme conditions (salinity, UV/radiation exposure, acidic/alkaline or high temperature, characteristics highlighted in Supporting Information Tables S2 and S3) were detected in both bacterial (e.g., *Rubrobacter, Deinococcus, Thermomicrobium, Gillisia, Acidimicrobium*) and archaeal communities (e.g., *Halomarina, Halobacterium*). Other genera indicated the capacity to autotrophically fix carbon (e.g., *Phormidium, Aurantiamonas, Thiobacillus, Thioclava, Rhodobacter, Acidimicrobium*), cycle nitrogen (e.g., *Malikia, Ochrobactrum, Nitrososphaera, Novosphingobium*) and sulfur (e.g., *Thioclava, Rhodovulum, Desulfuromonas*) and utilize minerals such as iron and manganese (e.g., *Aurantimonas*, *Acidimicrobium, Ferrimicrobium*). In addition, many of the detected genera have been isolated from or detected in marine systems (e.g., *Rubrobacter*, *Jannaschia, Maribius* and many others), which, given the close proximity of Portchester Castle to the sea (< 50 m), would suggest sea spray may contribute to a saline environment on the stone and act as a marine inoculum for these communities. Similarly in other low biomass systems, such external sources of nutrients are critical to microbial community structure and function (Logue *et al*., 2012).

**Figure 2:**
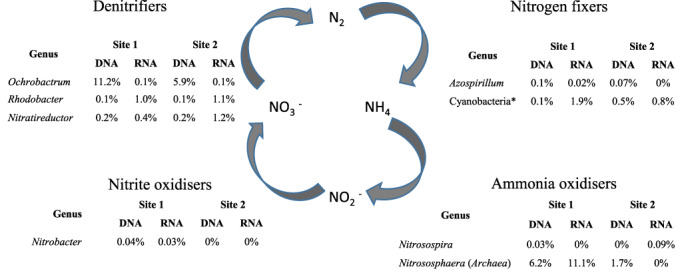
Reconstruction of the N‐cycle at sites 1 and 2 using the potential capability of the specific genera analyzed by 16S rRNA gene amplicon sequencing. *Nitrogen‐fixing cyanobacteria include *Calothrix, Chroococcidiopsis, Cyanothece, Leptolyngbya, Microcoleus, Nostoc, Scytonema* and *Synechococcus* at both sites.

The bacterial diversity shown at these sites raises the question whether these communities carry the capacity to be essentially self‐sustaining, as can be asked of other low‐biomass communities. To do so they would need to be capable of complete cycling of essential elements such a carbon, nitrogen and sulfur. To investigate whether complete elemental cycling is possible within these communities we have categorized detected genera into functional groups within the N‐cycle. By allocating a potential N‐cycling function to a number of the genera detected in the samples from sites 1 and 2 it is possible to reconstruct an almost complete nitrogen cycle for both sites just using 16S rRNA gene‐based data (Fig. 2; Supporting Information Tables S2 and S3). A wide range of genera capable of nitrogen fixation and denitrification as well as specific ammonia oxidizing genera have been detected (Fig. 2). There are very few 16S sequences from nitrite oxidisers such as *Nitrobacter*, with none detected at site 2. Given there was also no evidence of the bacteria linked to complete ammonia oxidation (comammox; Daims *et al*., 2015; Van Kessel *et al*., 2015) this represents the only break in the cycle.

**Figure 3:**
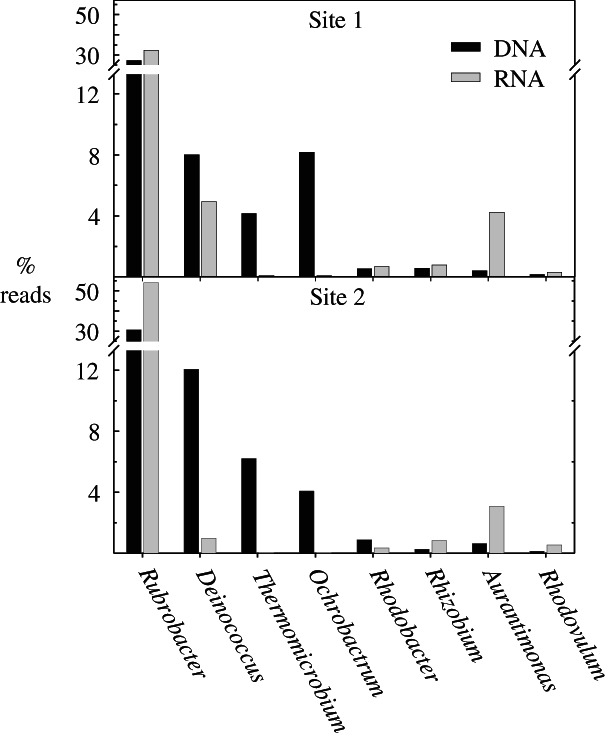
Comparison of presence (DNA) and relative activity (RNA) of bacterial genera detected on damaged stoneworks, Porchester Castle.

While we have identified organisms that may have the necessary enzymes to drive the nitrogen cycle this does not prove that these organisms are active or actually contain the relevant genes, such as *nirK* (nitrite reductase for denitrification) or *amoA* (ammonia monooxygenase for ammonia oxidation) that encode enzymes which drive these processes. Despite the very low RNA yield from these low biomass environmental samples an RT‐PCR analysis of 16S rRNA from sites 1 and 2 directly shows most of the N‐cycle organisms identified (Fig. 2) are also likely to be active in these samples (Fig. 3; Supporting Information Table S2 and S3). For ammonia oxidisers either presence (DNA) or expression (RNA) was detected by 16S rRNA analysis in both site 1 and site 2 samples.

The presence of functional genes for denitrification (bacterial *nirK*) and ammonia oxidation (bacterial *amoA*) was also analyzed from site 2, *nirK* by cloning and sequencing and *amoA* by both pyrosequencing and cloning and sequencing. Bacterial *nirK* from *Ochrobactrum, Azospirillum* and *Rhodobacter* were all detected (Supporting Information Fig. S2) while the *amoA* genes from *Nitrosospira* and from the archaea *Nitrososphaera* were detected in both cloned and pyrosequencing analyses (Supporting Information Figs. S3 and S4; Supporting Information Table S4). Bacterial ammonia oxidizing gene expression was also supported by an *amoA* RT‐PCR analysis at site 1, where *Nitrosospira*‐like *amoA* sequences were detected and dominated (Supporting Information Table S4), indicating this gene is expressed at this site despite the low abundance of *Nitrosospira* in the 16S rRNA gene libraries.

This analysis shows that the potential to perform an essentially complete nitrogen cycle exists at sites 1 and 2. For site 3, which has a distinct community compared with sites 1 and 2, it is also possible to reconstruct a N‐cycle, with potential for denitrification with *Ochrobactrum* and *Rhodobacter*, nitrogen fixation with *Chroococcidiopsis* and *Azospirillum*, ammonia and nitrite oxidation by *Nitrososphaera* and *Nitrobacter* respectively. Therefore, 16S rRNA gene analysis supports the potential for all 3 sites to cycle nitrogen; analysis of 16S rRNA directly at sites 1 and 2 indicates that the relevant genera are likely to be active and functional gene analysis supports the dominance of *Nitrosospira* and *Nitrososphaera* as ammonia oxidisers in these samples.

In addition to the N‐cycle there is clear evidence of potential for both phototrophic and non‐phototropic CO_2_ fixation in all three sites. Cyanobacteria are detectable, albeit at low relative abundances (0.02%–0.5%), at all three sites along with other carbon fixers such as *Aurantimonas, Acidimicrobium* and *Thiobacillus* (Supporting Information Table S2). The presence of autotrophic carbon fixation capacity was confirmed by the amplification of red RuBisCo genes linked to *Aurantimonas* (Supporting Information Fig. S5), which was detected by 16S rRNA analysis at all three sites (Supporting Information Table S2) and was detected more readily using RT‐PCR (sites 1 and 2) suggesting that *Aurantimonas* was likely to be active at these sites (Fig. 3). The presence of *Nitrobacter*, detected only very rarely in these samples by 16S rRNA, was confirmed by the detection of green RuBisCo genes closely related to it in site 2 (Supporting Information Fig. S6).

Sulfate is almost certainly freely available on these stones due to its close proximity to the sea and so, given that sulfate assimilation is a common microbial characteristic (Kredich, 1996), simple assimilation and mineralization processes could create a rudimentary sulfur cycle. However, the detection of genera with the capacity to oxidize sulfur, such as *Thioclava* and *Thiobacillus*, along with the detection of sulfur and sulfate reducing genera (*Desulfuromonas* and *Desulfotomaculum*; Supporting Information Table S2) indicate that a complete sulfur cycle may be functioning within these communities. It had been reported that sulfur oxidizing bacteria were not present on Northern European stoneworks (Warscheid and Braams, 2000), but the detection of a number of known sulfur oxidizing bacterial lineages here (including *Rhodovulum* and *Thioclava;* Supporting Information Table S2) suggests that these microorganisms may play a role in degradation processes at Portchester Castle. Sulfur oxidizing bacteria and fungi, which can produce sulfuric acid, have been isolated from degrading sandstone at Angkor Wat in Cambodia (Kusumi *et al*., 2011; Xu *et al*., 2018), highlighting the importance of considering how nutrient cycling may contribute to degradation processes. The detection of genera capable of utilizing manganese and iron as electron donors (*Aurantimonas*, *Acidimicrobium*) further indicates a potential for chemoautotrophy in these communities (Supporting Information Table S2). The additional capacity to oxidize common organic air pollutants such as aliphatics and aromatics (e.g., *Malikia, Pseudomonas, Alcanivorax*) could provide a further source of carbon to sustain these communities (Supporting Information Table S2) (Saiz‐Jimenez, 2003).

Therefore, using the organisms detected using 16S rRNA and functional gene analysis within these samples suggest that the microbial communities from the stones of Portchester Castle have the capacity to be complete, self‐sustaining ecosystems. Inorganic acids produced by sulfur oxidizing and nitrifying microbes can chemically attack stone materials leading alterations such as exfoliation and corrosion (Mansch and Bock, 1998). In unsheltered stonework areas the alteration appears as a pulverization (exfoliation seen in samples 1 and 2) while in sheltered areas that are protected from rain, dark‐black encrustations can develop (as seen in sample 3) (Warscheid and Braams, 2000; Ranalli *et al*., 2009). Production of nitrate, and therefore potentially nitric acid, by ammonia oxidizing bacteria and archaea have been suggested to contribute to the deterioration of calcareous sandstone (Meincke *et al*., 1989; Warscheid and Braams, 2000; Ranalli *et al*., 2009). Similarly, manganese and iron oxidation may exacerbate corrosion and alter pH dynamics in areas covered by the microbial encrustations. The potential presence of complete nitrogen and sulfur cycling in these systems would suggest that acidic conditions created by intermediates of these cycles, such as sulfur oxides, sulfide, nitrate and nitrite, probably contribute to the damage caused to the stonework (Mansch and Bock, 1998; Warscheid and Braams, 2000; Scheerer *et al*., 2009). It is reasonable to assume that microbial communities in other low‐biomass systems would require similar levels of diversity and functional capabilities to maintain a self‐sustaining community.

Here we show that the natural microbial communities from an important cultural heritage site in the UK are highly diverse and are potentially self‐sustaining ecosystems capable of cycling carbon, nitrogen and sulfur. It is reasonable to hypothesise that damage linked to these microbial communities is caused by the end‐products of essential ecosystem functions, such as nitrogen and sulfur cycling, as well as by direct degradation processes such as iron or manganese oxidation. Therefore, interventions that target essential ecosystem functions, compromising the functionality of the whole community, could be an effective approach to management of culturally important stoneworks.

## Supporting information


**Fig. S1.** Sampling sites at Portchester Castle. **A.** Richard II's palace (1390–1450). **B.** Samping sites, from a window. Sites 1 and 2 were exfoliated stone, Site 3 was a dark encrustation. Schematic of Richard II's palace reproduced with permission of Historic England.
**Fig. S2.** Neighbour‐joining tree of bacterial *nirK* clones from Portchester Castle, Site 2. Tree was rooted with the outgroup *Nitrosomonas europea* ATCC25978 (EF016124; not shown).
**Fig. S3.** Neighbour‐joining tree of bacterial *amoA* clones from Portchester Castle, Site 2. Tree was rooted with the outgroup *Nitrosococcus oceani* ATCC19707 (AF272521; not shown). Bootstrap values >50% are given at nodes.
**Fig. S4.** Neighbour‐joining tree of archaeal *amoA* clones from Portchester Castle, Site 2. Tree was rooted with the outgroup *Nitrosopumilus maritimus* SCM1 (EU239959; not shown). WWT = wastewater treatment plant. Bootstrap values >50% are given at nodes.
**Fig. S5.** Neighbour‐joining tree of Red RuBisCo (*cbbL*) clones from Portchester Castle, Site 2. Tree was rooted with the outgroup *Rhodobacter sphaeroides* 2R (AY450589; not shown). WWT = wastewater treatment plant. Bootstrap values >50% are given at nodes.
**Fig. S6.** Neighbour‐joining tree of Green RuBisCo (*cbbL*) clones from Portchester Castle, Site 2. Tree was rooted with the outgroup *Thialkalivibrio denitrificans* ALJD (AY914807; not shown). Bootstrap values >50% are given at nodes.

**Table S1.** PCR primers used in this study.

**Table S2.**

**Table S3.**

**Table S4.**
