# Supplementary material for: Nutrient cycling potential within microbial communities on culturally important stoneworks
Source: Environ Microbiol Rep. 2018 Dec 25;11(2):147–54. doi: 10.1111/1758-2229.12707 (PMC7379959; doi:10.1111/1758-2229.12707)
Supplement: Supplementary file 1 — Fig. S1. Sampling sites at Portchester Castle. A. Richard II's palace (1390–1450). B. Samping sites, from a window. Sites 1 and 2 were exfoliated stone, Site 3 was a dark encrustation. Schematic of Richard II's palace reproduced with permission of Historic England. Fig. S2. Neighbour‐joining tree of bacterial nirK clones from Portchester Castle, Site 2. Tree was rooted with the outgroup Nitrosomonas europea ATCC25978 (EF016124; not shown). Fig. S3. Neighbour‐joining tree of bacterial amoA clones from Portchester Castle, Site 2. Tree was rooted with the outgroup Nitrosococcus oceani ATCC19707 (AF272521; not shown). Bootstrap values >50% are given at nodes. Fig. S4. Neighbour‐joining tree of archaeal amoA clones from Portchester Castle, Site 2. Tree was rooted with the outgroup Nitrosopumilus maritimus SCM1 (EU239959; not shown). WWT = wastewater treatment plant. Bootstrap values >50% are given at nodes. Fig. S5. Neighbour‐joining tree of Red RuBisCo (cbbL) clones from Portchester Castle, Site 2. Tree was rooted with the outgroup Rhodobacter sphaeroides 2R (AY450589; not shown). WWT = wastewater treatment plant. Bootstrap values >50% are given at nodes. Fig. S6. Neighbour‐joining tree of Green RuBisCo (cbbL) clones from Portchester Castle, Site 2. Tree was rooted with the outgroup Thialkalivibrio denitrificans ALJD (AY914807; not shown). Bootstrap values >50% are given at nodes. [file EMI4-11-147-s001.pdf]

A. Richard II's palace (1390-1450)

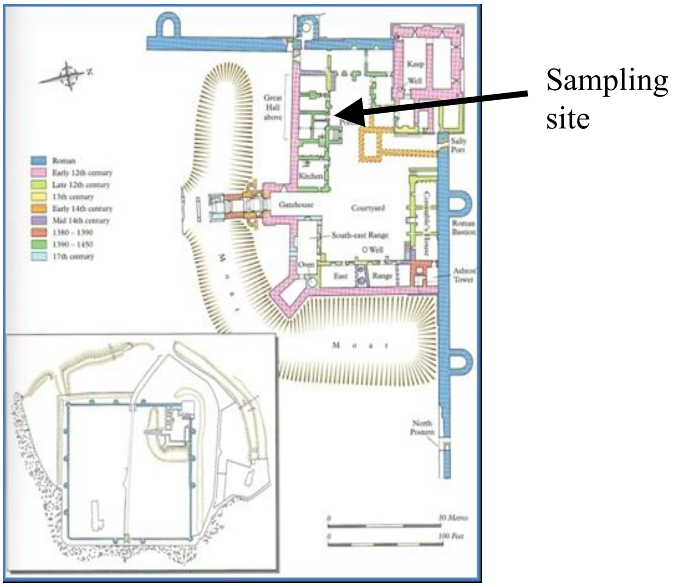

B. Sampling sites

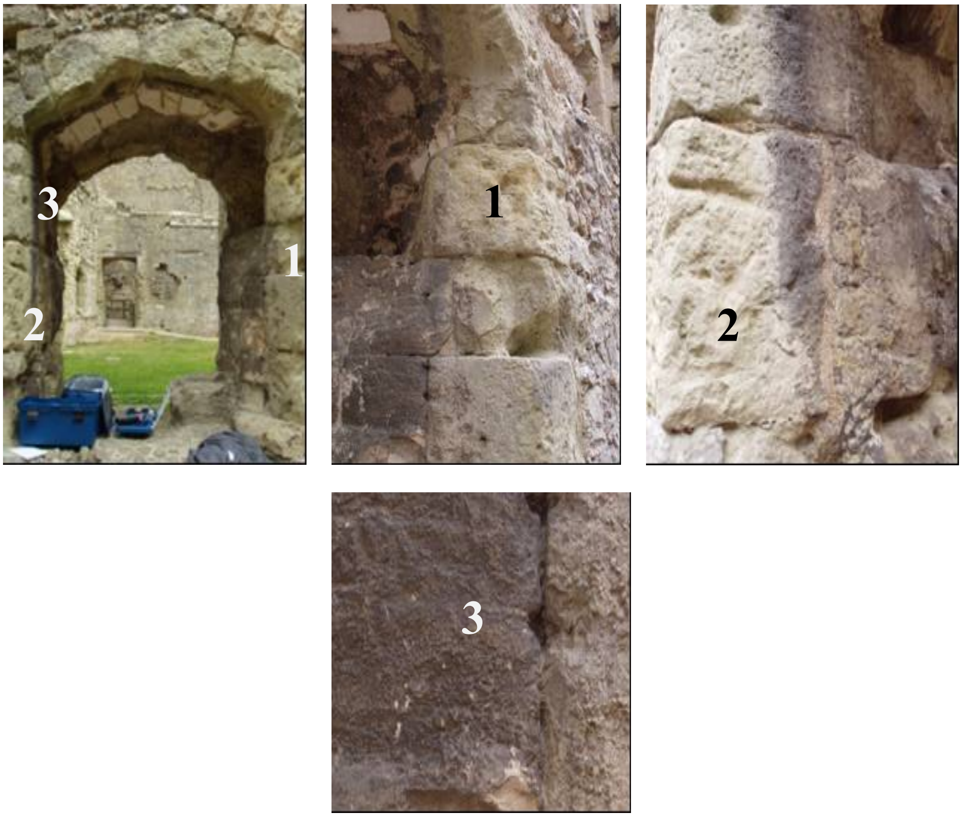

Figure S1. Sampling sites at Portchester Castle. **A.** Richard II's palace (1390-1450). **B.** Samping sites, from a window. Sites 1 and 2 were exfoliated stone, Site 3 was a dark encrustation. Schematic of Richard II's palace reproduced with permission of Historic England.

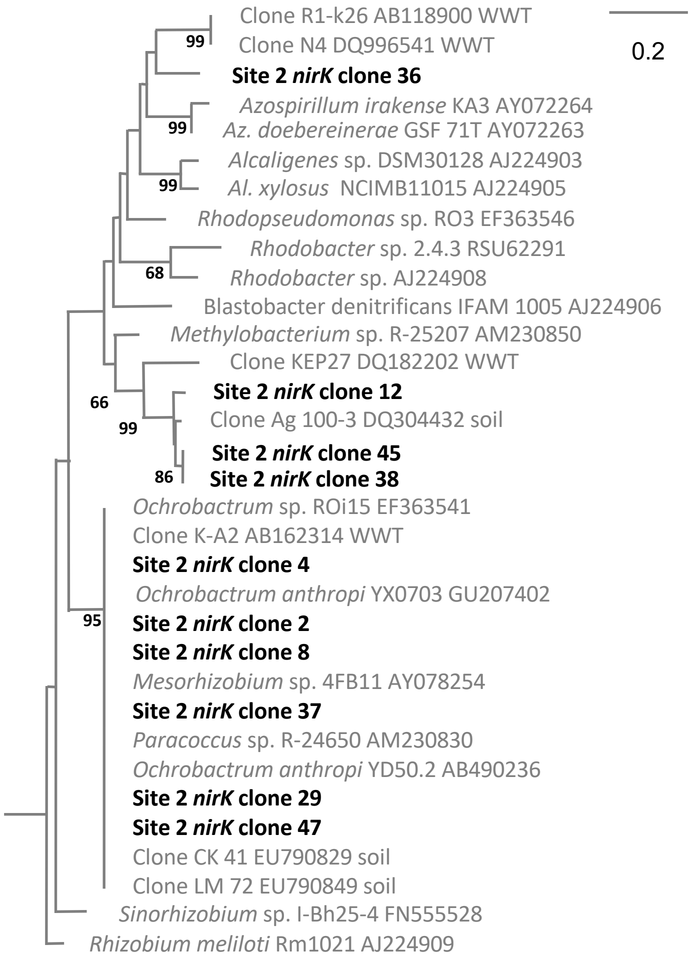

Figure S2. Neighbour-joining tree of bacterial *nirK* clones from Portchester Castle, Site 2. Tree was rooted with the outgroup *Nitrosomonas europea* ATCC25978 (EF016124; not shown).

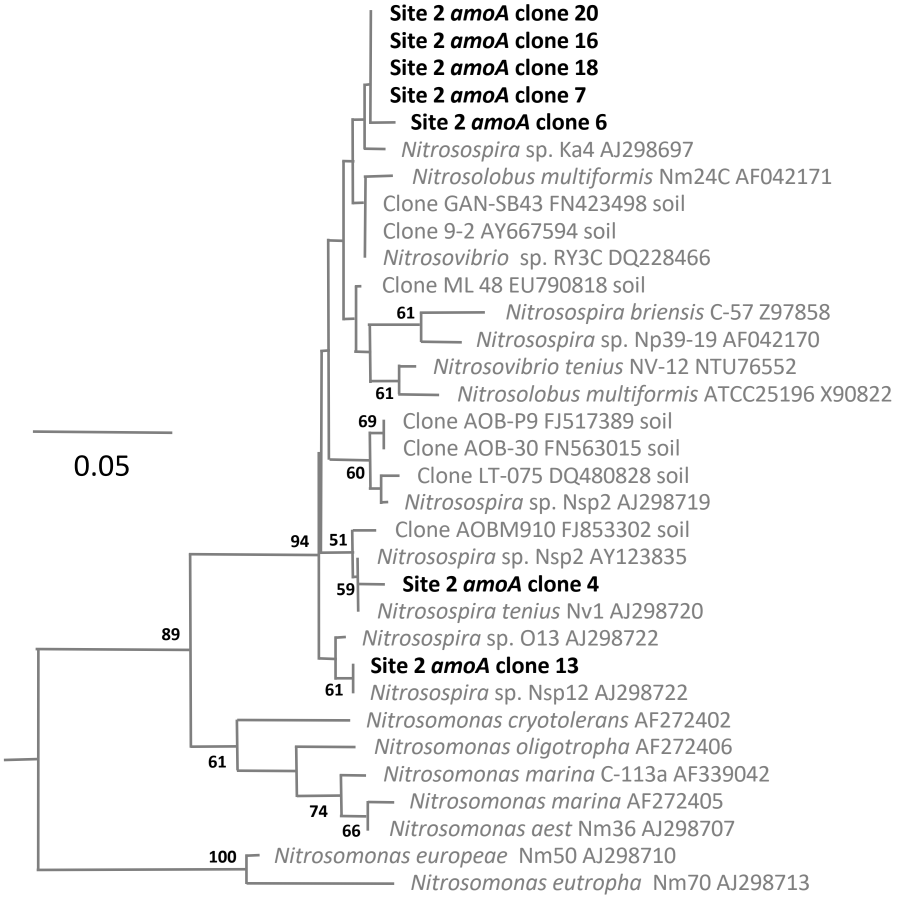

Figure S3. Neighbour-joining tree of bacterial *amoA* clones from Portchester Castle, Site 2. Tree was rooted with the outgroup *Nitrosococcus oceani* ATCC19707 (AF272521; not shown). Bootstrap values >50% are given at nodes.

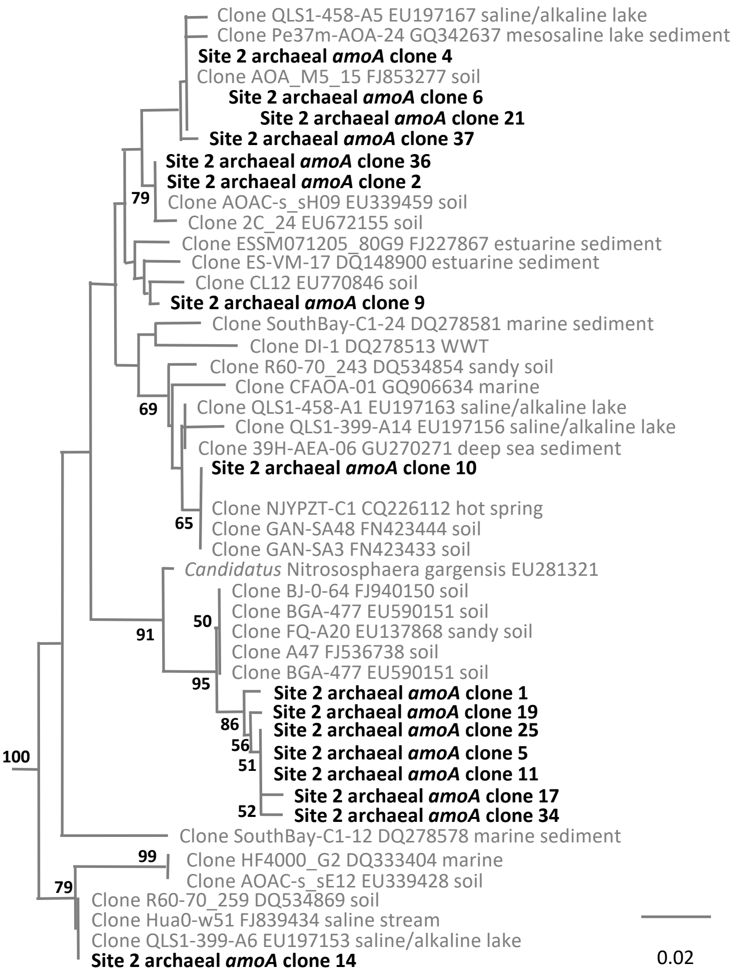

Figure S4. Neighbour-joining tree of archaeal *amoA* clones from Portchester Castle, Site 2. Tree was rooted with the outgroup *Nitrosopumilus maritimus* SCM1 (EU239959; not shown). WWT = wastewater treatment plant. Bootstrap values >50% are given at nodes.

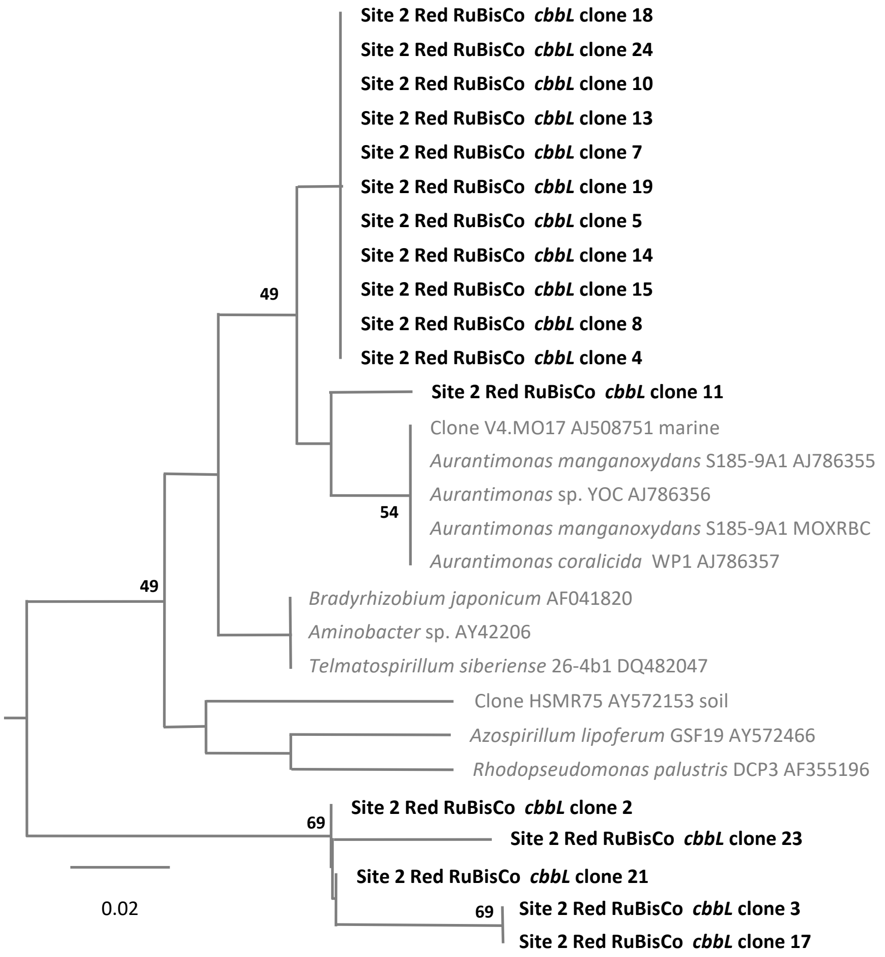

Figure S5. Neighbour-joining tree of Red RuBisCo (*cbbL*) clones from Portchester Castle, Site 2. Tree was rooted with the outgroup *Rhodobacter sphaeroides* 2R (AY450589; not shown). WWT = wastewater treatment plant. Bootstrap values >50% are given at nodes.

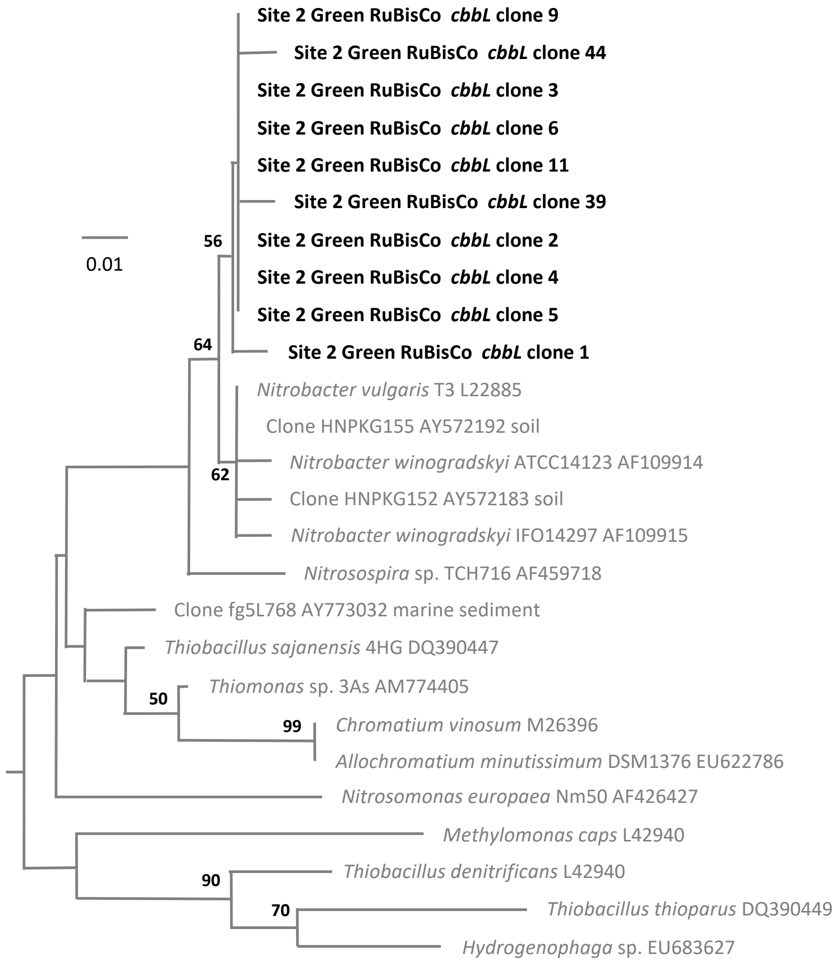

Figure S6. Neighbour-joining tree of Green RuBisCo (*cbbL*) clones from Portchester Castle, Site 2. Tree was rooted with the outgroup *Thialkalivibrio denitrificans* ALJD (AY914807; not shown). Bootstrap values >50% are given at nodes.
