# Supplementary material for: Nutrient cycling potential within microbial communities on culturally important stoneworks
Source: Environ Microbiol Rep. 2018 Dec 25;11(2):147–54. doi: 10.1111/1758-2229.12707 (PMC7379959; doi:10.1111/1758-2229.12707)
Supplement: Supplementary file 2 — Table S1. PCR primers used in this study. [file EMI4-11-147-s002.docx]

**Nutrient cycling potential within microbial communities that degrade culturally important stoneworks**

Elisabetta Zanardini, Eric May, Kevin J. Purdy and J. Colin Murrell

**Table S1.** PCR primers used in this study.

| **Primer pair** | **Length product** | **Target genes** | **References** |
| --- | --- | --- | --- |
| 341F-GC/907R | 550 bp | bacterial 16S rRNA | (Muyzer et al., 1993) |
| 21F-958R  Parch519f-Arch915R-GC | 400 bp | archaeal 16S rRNA | (Vissers et al., 2009) |
| amoA1F- amoA2R | 491 bp | bacterial *amoA* | (Rotthauwe et al., 1997) |
| Arch amoAF - Arch amoAR | 635 bp | archaeal *amoA* | (Francis et al., 2005) |
| nirK1F - nirK5R | 514 bp | *nirK* | (Braker et al., 1998) |
| cbbLR1F - cbbLR1R | 800 bp | RuBisCO, red form | (Selesi et al., 2005) |
| cbbLG1F- cbbLG1R | 1100 bp | RuBisCO, green form |  |

**References**

Braker, G., Fesefeldt, A., and Witzel, K.P. (1998) Development of PCR primer systems for amplification of nitrite reductase genes (*nirK* and *nirS*) to detect denitrifying bacteria in environmental samples. *Appl. Environ. Microbiol.* **64**: 3769-3775.

Francis, C.A., Roberts, K.J., Beman, J.M., Santoro, A.E., and Oakley, B.B. (2005) Ubiquity and diversity of ammonia-oxidizing archaea in water columns and sediments of the ocean. *Proc. Nat. Acad. Sci. U.S.A.* **102**: 14683-14688.

Muyzer, G., De Waal, E.C., and Uitterlinden, A.G. (1993) Profiling of complex microbial populations by Denaturing Gradient Gel Electrophoresis analysis of PCR-amplified genes coding for 16S rRNA. *Appl. Environ. Microbiol.* **59**: 695-700.

Rotthauwe, J.H., Witzel, K.P., and Liesack, W. (1997) The ammonia monooxygenase structural gene *amoA* as a functional marker: Molecular fine-scale analysis of natural ammonia-oxidizing populations. *Appl. Environ. Microbiol.* **63**: 4704-4712.

Selesi, D., Schmid, M., and Hartmann, A. (2005) Diversity of green-like and red-like ribulose-1,5-bisphosphate carboxylase/oxygenase large-subunit genes (*cbbL*) in differently managed agricultural soils. *Appl. Environ. Microbiol.* **71**: 175-184.

Vissers, E.W., Bodelier, P.L.E., Muyzer, G., and Laanbroek, H.J. (2009) A nested PCR approach for improved recovery of archaeal 16S rRNA gene fragments from freshwater samples. *FEMS Microbiol. Letts.* **298**: 193-198.
